# The Importance of Newborn Genetic Screening for Early Identification of *GJB2* and *SLC26A4* Related Hearing Loss

**DOI:** 10.1002/ohn.1188

**Published:** 2025-02-26

**Authors:** Emily R Wener, Sharon L Cushing, Blake C Papsin, Dimitrios J. Stavropoulos, Roberto Mendoza‐Londono, Nada Quercia, Karen A. Gordon

**Affiliations:** ^1^ Archie's Cochlear Implant Laboratory, Neuroscience & Mental Health, Hospital for Sick Children Toronto Ontario Canada; ^2^ Department of Otolaryngology‐Head and Neck Surgery University of Toronto, Hospital for Sick Children Toronto Ontario Canada; ^3^ Department of Laboratory Medicine and Pathobiology University of Toronto Toronto Ontario Canada; ^4^ Genome Diagnostics Paediatric Laboratory Medicine, Clinical and Metabolic Genetics, The Hospital for Sick Children Toronto Ontario Canada; ^5^ Division of Clinical & Metabolic Genetics, The Hospital for Sick Children Toronto Ontario Canada; ^6^ Department of Genetic Counselling, The Hospital for Sick Children Toronto Ontario Canada; ^7^ Department of Molecular Genetics, Temerty Faculty of Medicine University of Toronto Toronto Ontario Canada

**Keywords:** genetics, GJB2, hearing loss, pediatrics, screening, SLC26A4

## Abstract

**Objective:**

To assess the added benefit of newborn genetic screening for *GJB2* and *SLC26A4* variants in conjunction with newborn hearing screening.

**Study Design:**

Retrospective cohort study.

**Methods:**

Children with known variants of *GJB2* and *SLC26A4* were identified from 485 children with hearing loss who underwent testing with Next Generation Sequencing (NGS) between January 2015 and February 2018, prior to expanded screening for genetic variants and congenital CMV. Children with two pathogenic or likely pathogenic variants of *GJB2* or *SLC26A4* were considered to have genetic hearing loss. NGS genetic data were compared to variants included in the expanded genetic screen for all newborns in Ontario and newborn hearing screening results.

**Setting:**

Canadian tertiary pediatric hospital.

**Results:**

Thirty‐five children with *GJB2* and *SLC26A4‐*associated hearing loss were identified by NGS (n = 27 *GJB2*‐HL; n = 8 *SLC26A4*‐HL). Of these, 20 (57%) had been identified by newborn hearing screening (14/27 52% *GJB2*‐HL; 6/8 75% *SLC26A4*‐HL). Ten of the 20 (50%) would also have been identified by genetic screening if it had been available (9/14 64% *GJB2*‐HL; 1/6 17% *SLC26A4*‐HL). An additional 8 children with *GJB2* or *SLC26A4‐*associated hearing loss passed their newborn hearing screen but showed hearing loss later; three of these children (38%) would have been identified by newborn genetic screening (3/6 *GJB2*‐HL; 0/2 *SLC26A4*‐HL).

**Conclusion:**

Genetic and hearing screening modalities in Ontario's expanded newborn hearing screening program improve early identification of children with hearing loss including those at risk of being missed by hearing screening alone. This was most clear for children with *GJB2*‐hearing loss.

The aim of this study was to assess the added benefit of newborn genetic screening for *GJB2* and *SLC26A4* variants in conjunction with newborn hearing screening in a large cohort of children followed for hearing loss at a Canadian pediatric tertiary healthcare center.

Childhood hearing loss is common, affecting 1 to 2 per 1000 newborns,^1^, ^2^, ^3^ and is estimated to be attributable to genetic etiology in 50% of cases.^4^, ^5^ Variants in over 100 unique genes have been implicated in congenital hearing loss.^6^, ^7^ Mutations in *GJB2* and *SLC26A4* are common etiologies of genetic hearing loss in North American populations.^8^ Consequently, genetic screening may be an essential component in conjunction with current universal newborn hearing screening programs^9^ for early identification of children with hearing loss and their subsequent referral to appropriate resources for intervention to support hearing and language development.^10^

In 2019, the province of Ontario implemented a first of its kind universal newborn screening program for hearing loss risk factors in collaboration between Newborn Screening Ontario (NSO) and the Infant Hearing Program (IHP).^11^ This program provides hearing screening, genetic testing, and serological tests for congenital cytomegalovirus (cCMV) to identify children with hearing loss.

Data to monitor the efficacy of the expanded newborn hearing screening program are ongoing. In the present study, data available from children with known genetic hearing loss identified prior to the onset of the expanded risk factor for hearing loss screening program were compared with the screening program protocol to determine the potential added value of genetic screening for all newborns in our province.

## Methods

The present study included 35 children with *GJB2*‐related hearing loss (*GJB2*‐HL) and *SLC26A4*‐related hearing loss (*SLC26A4*‐HL) who were identified from a cohort of 485 children with permanent hearing loss through a high throughput next generation sequencing (NGS) panel including 80 hearing loss‐associated genes at a tertiary care pediatric centre between January 2015 and February 2018. The catchment area of this center spans the Greater Toronto Area. These children were followed in the Department of Otolaryngology, Head and Neck Surgery as well as audiology. A retrospective analysis of NGS results was approved by the hospital's Research Ethics Board, which adheres to the Tri‐Council Policy on Ethical Conduct for Research Involving Humans (Study #1000060541).

Genetic data were gathered from the Genome Diagnostics Laboratory at the hospital. Variants were classified according to American College of Medical Genetics and Genomics (ACMG) guidelines as pathogenic (PV), likely pathogenic (LP), of uncertain significance (VUS), likely benign, or benign.^12^ Children with two PV or LP variants of *GJB2* or *SLC26A4* were considered to have a genetic etiology of hearing loss. NGS data of these children were then compared to the list of 10 *GJB2* and 9 *SLC26A4* variants included in Ontario's universal expanded newborn screening program.

Ontario's original hearing screening program began in 2002 and focused on the use of otoacoustic emissions (OAEs) in average risk newborns and auditory brainstem response (ABR) in high risk newborns.^11^ Newborns with high risk for hearing loss are defined by any one of the following: APGAR score ≤3 at 5 minutes, birthweight ≤1000*g*, congenital diaphragmatic hernia, first degree relative with hearing loss identified by age 10, hypoxic ischemic encephalopathy, intraventricular hemorrhage, peri‐ventricular leukomalacia, persistent pulmonary hypertension of the newborn, ventilatory support with high frequency ventilation or inhaled nitric oxide, cleft palate, extracorporeal membrane oxygenation, hyperbilirubinemia requiring exchange, proven TORCH infection, or genetic syndromes associated with permanent hearing loss.^11^ Any child with an abnormal hearing screen, either unilaterally or bilaterally, is referred for diagnostic audiometric testing.^11^ Children with aural atresia or microtia, CHARGE syndrome or proven meningitis do not undergo audiometric screening and are instead referred directly to audiology for diagnostic testing.^11^ The expansion of this program in 2019 acknowledged genetic variants and cCMV infection as important and common risk factors for permanent hearing loss.^11^ To test for these, newborn dried bloodspots are screened for 10 *GJB2* mutations (c.35delG, c.235delC, c.167delT, c.71G>A, c.310_323del, c.139G>T, c.‐23 + 1G>A, c.231G>A, c.427C>T, c.269T>C) and 9 *SLC26A4* mutations (c.707T>C, c.1001+1G>A, c.1246A>C, c.919‐2A>G, c.2168A>G, c.1003T>C, c.1229C>T, c.1614+1G>A, c.1541A>G) using mass array technology.^13^, ^14^ A singular *GJB6* mutation (D13S1830 342 kb deletion, regulatory *GJB2* mutation) is reflexively screened for in children with at least one variant of *GJB2*.^13^ The aforementioned variants are highly penetrant, truncating mutations, conferring a high risk for permanent congenital hearing loss.^14^ The presence of two variants in the same gene is reported as a positive screen, and carrier status is disclosed by request only.^13^ Dried bloodspots are also screened for CMV using real‐time polymerase chain reaction (PCR) assay.^11^, ^14^ Any child with a positive genetic or CMV screen is referred for further genetic counseling and potential additional workup.^11^

The children studied here were screened using audiometric modalities alone, as they were all born prior to the implementation of universal genetic screening for hearing loss in 2019. The newborn hearing screening results of these children were reviewed to determine which children were successfully identified by newborn audiometric screening alone.

Children were grouped according to their genetic etiology of hearing loss (*GJB2*‐HL or *SLC26A4*‐HL), audiometric hearing screening result (referred, passed, or no screening data available). Clinical data, including age and degree of hearing loss at the time of hearing loss diagnosis, and age at the time of first cochlear implant (CI) surgery were compared between groups. Hearing loss diagnosis was defined as the time of definitive, diagnostic audiometric testing resulting in a diagnosis of hearing loss. Degree of hearing loss at diagnosis was quantified by pure tone average (PTA), calculated by an average of thresholds obtained at 0.5, 1, 2, and 4 kHz. By convention, no response to maximal stimulus intensity at a given frequency was documented as a threshold of 130 dB HL. The projected genetic screening result (refer or pass) was determined by comparing the variants observed in our cohort to those included in the genetic screen. The projected genetic refer group included children with two variants of *GJB2* or *SLC26A4* that appear in the genetic screen. All other children were included in the projected genetic pass group. As many of the children went on to receive CIs, the age at first CI surgery was assessed.

### Statistical Analysis

Linear mixed‐effects regression analyses were conducted using the lmer4 package (Douglas Bates) and RStudio, version 1.4.1717 (RStudio Inc).^15^ Outcomes were: (1) age at hearing loss diagnosis, (2) degree of hearing loss at diagnosis, quantified by pure tone average (PTA) in dB HL, and (3) age at first CI surgery. Fixed effects were sex (male or female), group (*GJB2*‐HL or *SLC26A4*‐HL), hearing screening result (referred, passed, or no data available), ear (better or poorer), and projected genetic screening result (refer or pass). A random intercept for each participant was included in each analysis. The means and confidence intervals estimated from the model are reported in the text and tables. Statistical significance was set at *P* < .05.

## Results

**Figure 1:**
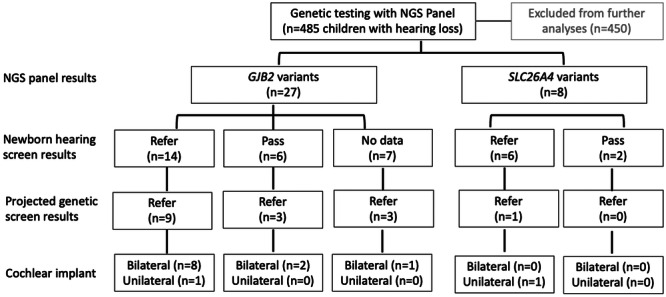
Flowchart of cohort by Next Generation Sequencing panel results, newborn hearing results, projected genetic screen results, and ultimate use of cochlear implants.

As shown in the flowchart in Figure 1, NGS testing of the total cohort (n = 485, M:F = 250:235) revealed 35 children (M:F = 11:24) with *GJB2* (n = 27, M:F = 9:18) and *SLC26A4* (n = 8, M:F = 2:6) associated hearing loss. None of these children were found to have congenital CMV infection. Children were mean (SD) = 5.5(6.1) years of age at the time of genetic testing. Thirty‐three children had bilateral hearing loss (*GJB2* = 25, *SLC26A4* = 8) and two children had unilateral hearing loss (*GJB2* = 2, *SLC26A4* = 0) at the time of genetic testing. Sixteen children would have been referred based on present genetic screening protocols. Most (23/35, 66%) received cochlear implants (CIs) either bilaterally (n = 17, *GJB2* = 13, *SLC26A4* = 4) or unilaterally (n = 6, *GJB2* = 2, *SLC26A4* = 4). The average age at the time of first cochlear implant surgery was mean (SD) = 3.4(2.6) years (*GJB2* = 2.3 (1.6) years, *SLC26A4* = 5.3 (3.1) years).

### Multiple Variants Contribute to GJB2 and SLC26A4‐Associated Hearing Loss

The unique variants identified by Next Generation Sequencing (NGS) in children with *GJB2* and *SLC26A4*‐HL are detailed in Table 1. The variants included in the current genetic screening protocol for risk factors of hearing loss in Ontario are highlighted in bold. Of the 15 *GJB2* variants found, 7 (47%) are included in the newborn genetic screen. Another 3 *GJB2* variants are included in the newborn genetic screen but did not appear in the present cohort (c.310_323del, c.139G>T, c.427C>T). Of the 13 unique *SLC26A4* variants identified through NGS, 3 (23%) are included in the current genetic screening protocol. There are six other *SLC26A4* variants that appear in the screening protocol but none of these (c.1246A>C, c.2168A>G, c.1003T>C, c.1229C>T, c.1614+1G>A, c.1541A>G) were found in this cohort through NGS.

**Table 1:** Unique *GJB2* and *SLC26A4* Variants Identified by NGS

*GJB2* Variants	*SLC26A4* variants
c.35del	c.2T>A
c.313_326del	c.412G>T
**c.35delG**	c.626G>T
**c.231G**>**A**	**c.1001 + 1G**>**A**
c.167del	c.1268delC
c.101T>C	c.2228T>A
**c.235delC**	c.269C>T
**c.−23** + **1G**>**A**	c.2086C>T
**c.167delT**	**c.919‐2A**>**G**
c.109G>A	**c.707T**>**C**
**c.269T**>**C**	c.1708G>A
c.298C>T	c.1614+1G>T
c.1A>G	c.2145G>T
**c.71G**>**A**	
c.257C>G	

All variants listed are classified as pathogenic (PV) or likely pathogenic (LP) according to American College of Medical Genetics and Genomics guidelines. Variants identified in our cohort that also appear on our province's newborn screening protocol^13^ are highlighted in bold.

### Addition of Genetic Screening Improves Hearing Loss Identification by 9%

As shown in Figure 1, newborn audiological screening identified hearing loss in 20/35 (57%) of children in this cohort (14/27 52% *GJB2*‐HL; 6/8 75% *SLC26A4*‐HL) and all 20 children were referred based on findings in both ears. Of the children identified by newborn hearing screening, 10/20 (50%) would have also been identified by genetic screening (9/14 64% *GJB2*‐HL; 1/6 17% *SLC26A4*‐HL) if available at that time, highlighting the redundancy afforded by the combination of genetic and audiometric screening modalities. Three children who passed their newborn audiometric hearing screen would have been identified by the current newborn genetic screening protocol (3/6 50% *GJB2*‐HL; 0/2 0% *SLC26A4*‐HL), thereby increasing the potential rate of identification of hearing loss in newborns to 66% (23/35). Newborn hearing screening data were unavailable for seven children (seven *GJB2*‐HL; zero *SLC26A4*‐HL) and three (43%) would have been identified by genetic screening. Two of these seven children (29%) were born outside of our province, so they did not undergo hearing screening whereas the remaining five children (71%) either did not undergo screening despite being born in Ontario or their screening data could not be found by retrospective chart review.

### Children With GJB2 and SCL26A4 Related Hearing Loss are Identified Earliest if Referred on Newborn Hearing Screen

**Figure 2:**
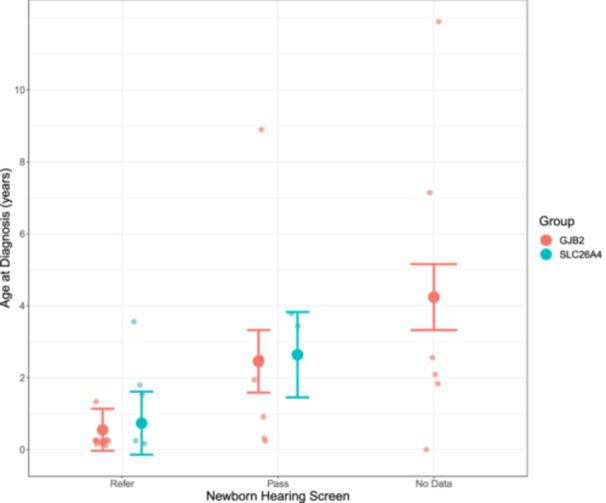
Children who referred on their hearing screen were diagnosed with hearing loss at younger ages than those who passed or were not screened (*P* = .0024). This was not affected by genetic group (*GJB2* vs *SLC26A4*, *P* = .89).

Effects of hearing screen on age at diagnosis, shown in Figure 2, were significant (*F*_2,28_ = 7.57, *P* = .0024) and confirm the benefits of effective hearing screening for both children with *GJB2* and *SLC26A4* associated hearing loss. Age at hearing loss identification through diagnostic audiology was 0.64 (−0.48 to 1.76) years in referred children whereas those children who passed the hearing screen were not diagnosed with hearing loss until 2.55 (0.69–4.40) years old. Children who were not screened were identified at even older ages 4.33 (2.17‐6.49) years. There was no effect of genetic group on age at diagnosis of hearing loss (*F*_1,28_ = 0.021, *P* = .89). There was no interaction between genetic group and hearing screening result (*F*_1,27_ = 0.0003, *P* = .99).

### Genetic Group Affects Identification of Newborn Hearing Loss, Degree of Hearing Loss, and Age at Cochlear Implantation

**Figure 3:**
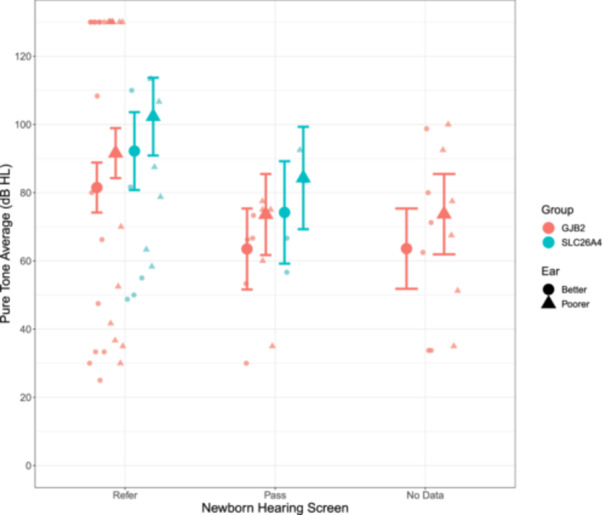
Children who referred on their newborn hearing screen had poorer hearing at diagnosis compared to those who passed (*P* = 0.24). There was a significant difference in hearing between ears (*P* < 0.001) across genetic groups.

As shown in Figure 3, children who were referred based on the newborn hearing screen had a wide range of hearing loss in both ears at diagnosis (range: 25‐130 dB HL) whereas children who passed their screen, or did not undergo screening had moderately‐severe to severe hearing loss. There was no significant difference in hearing thresholds based on screening results (*F*_2,28_ = 1.52, *P* = .24). There was no significant difference in degree of hearing loss at diagnosis between children with *SLC26A4*‐HL and children with *GJB2*‐HL (*F*_1,28_ = 0.69, *P* = 0.41) and no significant interaction between genetic group and hearing screening results (*F*_1,28_ = 0.96, *P* = .34). There was a slight but significant difference in hearing thresholds between ears (*F*_1,32_ = 22.03, *P* < .001).

**Figure 4:**
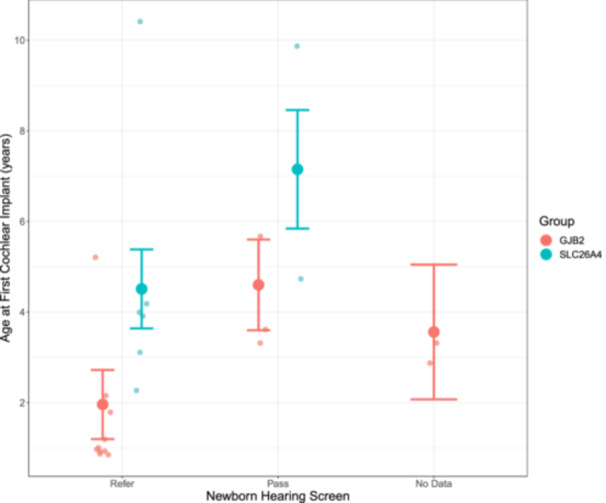
Children who referred on their hearing screen were implanted significantly earlier than those who passed their hearing screen (*P* = .037). Children with *GJB2* variants who referred on their newborn screen were the earliest implanted (*P* = .90).

Effects of screening data and genetic group on age at first cochlear implant surgery are shown in Figure 4. Children identified by newborn hearing screening underwent their first cochlear implant surgery significantly earlier (*F*_2,17_ = 4.03, *P* = .04) than those who passed their newborn hearing screen and those who did not undergo screening (refer: 3.24 (2.14‐4.33) years old at surgery, pass: 5.88 (3.81‐7.94) years old at surgery, not screened 4.83 (1.33‐8.34) years old at surgery). Children with *GJB2*‐HL were implanted at significantly younger ages than children with *SLC26A4*‐HL (*F*_1,17_ = 11.90, *P* = 0.003). There was no significant interaction between screening results and genetic group (*F*_1,16_ = 0.015, *P* = .90) suggesting that there are independent delays related to the hearing screen result and the type of genetic hearing loss.

## Discussion

In the present study, 57% of 35 children with *GJB2* and *SLC26A4* hearing loss were identified by newborn hearing screening with audiometric testing alone. Half of the children with *GJB2* hearing loss missed by newborn hearing screening would have been identified by genetic screening, thereby increasing the potential rate of identification to 66%.

The importance of newborn hearing screening was confirmed, as children identified by newborn screening were diagnosed with a wide range of hearing loss significantly earlier than those whose eventual hearing loss was missed by the newborn hearing screening or those who did not undergo screening. These children received cochlear implants at younger ages (Figure 2).

It is well‐established that universal newborn hearing screening is essential to the early detection and diagnosis of hearing loss,^16^ with the goal of meeting the 1‐3‐6 paradigm, defined as screen by 1 month, diagnose by 3 months, and act by 6 months^9^ (p. 20).^11^, ^17^, ^18^ Indeed, following such paradigm has been shown to optimize speech and language outcomes in children with hearing loss.^9^ Early intervention allows children with hearing loss to achieve language on par with their normal hearing peers.^19^

Physiologic screening of auditory function using otoacoustic emissions and/or auditory brainstem responses provides fast, non‐invasive, low‐risk, inexpensive methods to identify hearing loss at birth.^20^, ^21^, ^22^ These measures are sensitive to hearing loss ranging from mild to profound,^23^ which is consistent with the wide range of hearing thresholds at diagnosis in children who were referred based on the hearing screen (Figure 3). Yet, physiologic screening tests do not identify children at risk of developing hearing loss nor provide any information related to the etiology of deafness.^19^ This leaves a gap in present screens based on auditory function at birth alone, with consequences for diagnosis and intervention.^19^ Some children with *GJB2* and *SLC26A4* related hearing loss passed their newborn hearing screen, suggesting the potential for both types of hearing loss to progressively deteriorate over time. Children with *SLC26A4* showed longer delays to cochlear implantation regardless of hearing screen result; this might reflect a slower rate of progressive hearing loss into cochlear implant candidacy range and/or lags in identification of progression with present audiological monitoring timelines. In this context, genetic hearing screening have improved the ability to monitor and adjust treatment in children with either *GJB2* or *SLC26A4* mutations.

Gaps in present hearing screening programs could be filled by adding genetic and congenital CMV testing,^24^ but protocols for genetic screening remain to be defined. Genetic screening adds expense to present hearing screening programs and thus needs to be assessed for cost‐effectiveness.^8^, ^24^ The mutations presently screened in Ontario would have increased identification of children with genetic hearing loss, but would not have identified all children in this cohort (Figure 1). A broader genome screening could improve identification of hearing loss associated variants in diverse populations, but this would come with increased cost and identification of variants of uncertain significance (VUS).^24^ Genetic screening might also be prone to regional differences which are not clear, given known ethnic biases in our knowledge of genetic causes of hearing loss.^8^, ^24^, ^25^ Potential solutions have been suggested, including genetic variant screening according to degree of hearing loss and/or ethnicity.^26^

Presently, it is clear comprehensive screening which incorporates tests of genetics, congenital CMV infection, and physiologic tests of auditory function has been recommended by clinical practice guidelines since 2019.^9^

### Potential Limitations

This study included children who did not undergo genetic screening at birth, but rather underwent definitive genetic testing in the context of a known hearing loss. Whereas potential genetic screening results were assessed, it will be important to determine the effectiveness of the genetic screening program prospectively. Moreover, the addition of congenital CMV screening was not assessed and is also likely to play an important role in increasing identification of hearing loss and/or the risk of future hearing loss in newborns. Finally, our ability to comment on the ethnic similarity between our cohort and the broader population of our province is limited by the absence of self‐reported ethnicity data in referrals or followup. Our cohort was derived from the Greater Toronto Area, which represents the most diverse geographic area of the province. As a result, the studied cohort may have been more diverse than the general population.

## Conclusion

The combination of genetic and physiologic hearing screening in newborns had the potential to increase identification rates from 57% to 66% in a cohort of children with *GJB2* and *SLC26A4* hearing loss. Children identified by newborn hearing screening are diagnosed with hearing loss earlier than their peers who did not undergo screening or passed their screen. The provincial expanded Newborn Screening Program has good overlap with genetic findings in our clinical population with *GJB2*‐related hearing loss but captures only a small proportion of children in this region with variants in *SLC26A4*. Future studies of children who underwent universal screening with genetic, infectious, and physiologic hearing screening will aid in better understanding the utility of the screening program and guide future modification to meet the needs of the population.

## Author Contributions

**Emily R. Wener**, data collection, analysis, literature review, manuscript write up, figures; **Sharon L. Cushing**, concept, REB submission, analysis, manuscript write up, figures; **Blake C. Papsin**, concept, REB submission, manuscript write up, figures; **Dimitrios J. Stavropoulos**, concept, manuscript write up, figures; **Roberto Mendoza‐Londono**, concept, manuscript write up, figures; **Nada Quercia**, concept, analysis, manuscript write up, figures; **Karen A. Gordon**, concept, REB submission, analysis, manuscript write up, figures.

## Disclosures

### Competing interests

D.J.S. has equity in PhenoTips. No other conflicts to disclose.

### Funding source

None.
